# Current usage of inclisiran for cardiovascular diseases: overview of current clinical trials

**DOI:** 10.3389/fphar.2025.1449712

**Published:** 2025-02-14

**Authors:** Maan H. Harbi

**Affiliations:** Pharmacology and Toxicology Department, College of Pharmacy, Umm Al-Qura University, Makkah, Saudi Arabia

**Keywords:** cardiovascular diseases, low density lipoprotein, inclisiran, clinical trials, pcsk9, atherosclerotic cardiovascular disease, coronary artery disease

## Abstract

**Background:**

Cardiovascular diseases are predominant health conditions across the world due to their rising prevalence and association with several disorders. Inclisiran, a small interfering RNA (siRNA) therapy, lowers low density lipoproteins cholesterol (LDL-C) by targeting proprotein convertase subtilisin/kexin type 9 (PCSK9). Its exact role in cardiovascular diseases is not fully understood.

**Aim:**

This review examines current usage of Inclisran for cardiovascular diseases.

**Method:**

A detailed search of Clinicaltrials.gov was conducted to identify relevant studies that investigated heart diseases using Inclisran. Data on study design, sample size, intervention details, and outcomes related to Inclisran were extracted and analyzed.

**Results:**

As of 30 December 2024, there were 92 clinical trials on involving inclisiran found at clinicaltrials.gov. The investigation focused on studies that used inclisiran for cardiovascular diseases and found that limited clinical trials were identified with limited interventional measures. The final number of analyzed trials was 11. The follow-up duration ranged from 270 to 1,695 days with a total of 214,176 participants with a favorable safety profile and twice-yearly dosing after initial loading dose. The collective findings from these trials demonstrated effective LDL-C and PCSK9 lowering compared to baseline measurements. Most studies focused on LDL-C lowering rather than measuring cardiovascular outcomes.

**Conclusion:**

Although the studies showed inclisiran to lower LDL-C effectively, the evidence is still limited with regards to cardiovascular outcomes data. There is a need for real world studies addressing long-term safety, adherence and cost-effectiveness and therapeutic outcomes of combination therapy.

## Introduction

Cardiovascular disease (CVD) is a health concern globally contributing significantly to illness and death on a scale, including in the Middle East (Lippi and Sanchis-Gomar, 2020; Diez and Butler, 2023). Each year CVD is responsible for an estimated 17.9 million deaths (World Health Organization, 2023). It is linked to conditions such as heart disease, heart attacks, strokes and ischemic heart disease (Nabel, 2003; Rehman et al., 2021). Particularly noteworthy is that heart disease and strokes account for over 80% of all CVD related deaths with a troubling one-third occurring in individuals under 70 years old (Tsao et al., 2022).

Multiple behavioral risk factors play a substantial role in the development of heart disease. Excessive dietary consumption of saturated fat, transaturated fat, salt and sugar can increase risk of obesity, increased blood pressure and high levels of cholesterol. The increased risk of these are considered important causes of cardiovascular disease (Anand et al., 2015; Kazemi et al., 2022). Sedentary lifestyles, which involve minimal physical exercise and lengthy periods of sitting, can impair the heart and circulatory system over time (Leon-Latre et al., 2014; Same et al., 2016). Behavioral risk factors such as smoking and chronic stress can trigger inflammation, alter normal physiology of blood vessels and raise blood pressure (Ambrose and Barua, 2004; Duncan et al., 2019; Franklin et al., 2021). In order to prevent and treat heart disease, it is essential to address these risk factors through behavioral treatments such as stress management, regular exercise, good diet, and quitting smoking.

Recent years have seen the exploration of treatments to combat diseases through innovative therapies such, as monoclonal antibodies targeting proprotein convertase subtilisin/kexin type 9 (PCSK9) and strategies aimed at reducing low density lipoprotein cholesterol (LDL-C) levels effectively (Wong and Shapiro, 2019; Lippi and Sanchis-Gomar, 2020; Diez and Butler, 2023). Lowering LDL-C levels has been shown to bring benefits by decreasing the risk of heart attacks, enhancing function, reducing vessel wall inflammation and stabilizing atherosclerotic plaques (Douglas and Channon, 2014). PCSK9 is an enzyme that acts as a regulator of cholesterol levels by binding to LDL receptors on the surface of hepatocytes (Lagace, 2014; Hajar, 2019; Rimbert et al., 2021). These LDL receptors play an important role in the uptake of LDL-C from the ciruculation. However, the binding of PCSK9 to LDL receptors causes the LDL receptors to degrade and thus less LDL is cleared from the circulation (Lagace, 2014). Consequently, more LDL is accumulated in blood which significantly increases the risk of CVD (Yang et al., 2020). For this reason, PCSK9 is a considered a promising target for lowering LDL-C (Shapiro et al., 2015). A novel strategy for targeting PCSK9 with RNA based therapies has emerged in the past several years (Ickenstein and Garidel, 2019; Gareri et al., 2022). In this scenario inclisiran, a first-in-class small interfering RNA (siRNA) designed to specifically break down messenger RNA (mRNA) responsible, for PCSK9 has shown great potential in treating high cholesterol in patients with a high risk of CV events (Fitzgerald et al., 2014). Reduced PCSK9 levels allows LDL receptors to remain active on hepatocyte surfaces, hence promoting LDL-C removal from the circulation (Wang and Tall, 2017).

### Aims

To review and summarize the clinical trials pertaining to the use and effectiveness of inclisiran in cardiovascular diseases and to discuss its potential for long-term cardiovascular risk reduction.

## Methodological framework and research design

### Search strategy and inclusion criteria

On 30 December 2024, the ClinicalTrials.gov database was searched for clinical trials involving inclisiran to and including 30th December 2024. The keywords for condition/disease were; Cardiovascular diseases, Heart diseases, Coronary artery disease, Major adverse cardiovascular events, MACE, Cardiovascular outcomes, Atherosclerotic Cardiovascular Disease and atherosclerotic plaque. Inclisiran was fixed in the “other terms” search engine. To qualify, Completed and any phase clinical trials that included inclisiran as an intervention.

### Data extraction

From the results reported in the ClinicalTrials.gov registry, data were manually retrieved, and analyzed, according to the following elements. Study type: interventional/observational. Conditions: Trial design: All phases. Location: Open to any location. Study status: Completed and no longer looking for participants. A summary of the characteristics of the trials was obtained from the database, including the study title, NCT number, acronym, study status, conditions, interventions, primary outcome measure, study design, masking, phase, duration and number of patients enrolled are presented in Table 1.

**TABLE 1:** Summary of extracted clinical trials characteristics.

Serial No	NCT number	Study title	Acronym/study status	Brief summary	Conditions	Interventions
1	NCT02597127	Trial to Evaluate the Effect of ALN-PCSSC treatment on low density lipoprotein cholesterol (LDL-C)	ORION-1/completed with results	This study is a Phase II, placebo-controlled, double-blind, randomized trial in 480 participants with atherosclerotic cardiovascular disease (ASCVD) or ASCVD-risk equivalents and elevated LDL-C despite maximum tolerated dose of LDL-C lowering therapies to evaluate the efficacy, safety, and tolerability of inclisiran injection(s)	Atherosclerotic cardiovascular disease| familial hypercholesterolemia| Diabetes	DRUG: ALN-PCSSC | DRUG: Normal Saline
2	NCT03060577	An extension trial of inclisiran in participants with cardiovascular disease and high cholesterol	ORION-3/completed with results	This clinical study was designed to assess the efficacy, safety, and tolerability of long-term dosing of inclisiran and evolocumab given as subcutaneous injections in participants with high cardiovascular risk and elevated low-density lipoprotein cholesterol (LDL-C)	Atherosclerotic cardiovascular Disease| Symptomatic Atherosclerosis| Type2 Diabetes| Familial Hypercholesterolemia	DRUG: inclisiran |DRUG: evolocumab
3	NCT03814187	Trial to assess the effect of long term dosing of inclisiran in subjects with high CV risk and elevated LDL-C	ORION-8/Completed with results	The purpose of this extension study was to evaluate the efficacy, safety, and tolerability of long-term dosing of Inclisiran. The study was a global multicenter study	ASCVD| Elevated Cholesterol| Heterozygous Familial Hypercholesterolemia Homozygous Familial Hypercholesterolemia	DRUG: Inclisiran Sodium
4	NCT03399370	Inclisiran for Participants With Atherosclerotic Cardiovascular Disease and Elevated Low-density Lipoprotein Cholesterol	ORION-10/Completed with results	This is a Phase III, placebo-controlled, double-blind, randomized study in participants with ASCVD and elevated LDL-C despite maximum tolerated dose of LDL-C lowering therapies to evaluate the efficacy, safety, and tolerability of subcutaneous (SC) inclisiran injection(s). The study will be a multicenter study in the United States	ASCVD| Elevated Cholesterol	DRUG: Inclisiran Sodium| DRUG: Placebo
5	NCT03400800	Inclisiran for Subjects With ASCVD or ASCVD-Risk Equivalents and Elevated Low-density Lipoprotein Cholesterol	ORION-11/Completed with results	This is a Phase III, placebo-controlled, double-blind, randomized study in participants with ASCVD or ASCVD-Risk equivalents and elevated LDL-C despite maximum tolerated dose of LDL-C lowering therapies to evaluate the efficacy, safety, and tolerability of subcutaneous (SC) inclisiran injection(s). The study will be an international multicenter study (non-United States)	ASCVD| Risk Factor, Cardiovascular| Elevated Cholesterol	DRUG: Inclisiran Sodium| DRUG: Placebo
6	NCT04666298	Study of efficacy and safety of inclisiran in japanese participants with high cardiovascular risk and elevated LDL-C	ORION-15/completed with results	This was a placebo-controlled, double-blind, randomized trial in Japanese participants with history of coronary artery disease (CAD) or participants categorized in ‘high risk’ by JAS 2017 guideline, or Japanese participants with heterozygous familial hypercholesterolemia (HeFH) and elevated Low-density lipoprotein cholesterol (LDL-C) despite maximum tolerated dose of statin(s) to evaluate the efficacy, safety, tolerability, and PK of subcutaneous inclisiran injection(s)	Hypercholesterolemia Heterozygous Familial Hypercholesterolemia	DRUG: Inclisiran sodium| DRUG: Placebo
7	NCT04929249	A Randomized Study to Evaluate the Effect of an “Inclisiran First” Implementation Strategy Compared to Usual Care in Patients With Atherosclerotic Cardiovascular Disease and Elevated LDL-C Despite Receiving Maximally Tolerated Statin Therapy (VICTORION-INITIATE)	V-INITIATE/Completed with results	The purpose of this study was to assess the effectiveness of an “inclisiran first” implementation strategy (addition of inclisiran to maximally tolerated statin therapy immediately upon failure to achieve acceptable LDL-C with maximally tolerated statin therapy alone) compared to usual care in an atherosclerotic cardiovascular disease (ASCVD) population	Atherosclerotic Cardiovascular Disease	DRUG: Inclisiran
8	NCT04873934	Management of LDL-cholesterol With Inclisiran + Usual Care Compared to Usual Care Alone in Participants With a Recent Acute Coronary Syndrome	V-INCEPTION/Completed	The purpose of this study is to study the effectiveness of implementation of a systematic LDL-C management pathway including treatment with inclisiran in participants who have experienced a recent acute coronary syndrome (ACS) and have an increased LDL-cholesterol (>70 mg/dL) despite being treated with a statin drug	Acute Coronary Syndrome	DRUG: Inclisiran
9	NCT06507852	A Real-world Study of Inclisiran Adherence, Treatment Patterns, Patient Characteristics, and Effectiveness in ASCVD Patients With Hypercholesterolemia, ASCVD-risk Equivalent Patients With Hypercholesterolemia and Familial Hypercholesterolemia	NA/Completed	This was a descriptive, non-interventional, retrospective cohort study among patients with atherosclerotic cardiovascular disease (ASCVD) and hypercholesterolemia, ASCVD-risk equivalent (ASCVD-RE) or familial hypercholesterolemia (FH) administered inclisiran in a real-world setting in Austria	Atherosclerotic Cardiovascular Disease| Hypercholesterolemia	DRUG: Inclisiran
10	NCT04807400	Study in Primary Care Evaluating Inclisiran Delivery Implementation + Enhanced Support	SPIRIT/Completed with results	The purpose of this study was to evaluate the implementation of inclisiran in a regional primary care setting in the UK	Atherosclerotic Cardiovascular Disease| Atherosclerotic Cardiovascular Disease Risk Equivelents| Elevated Low Density Lipoprotein Cholesterol	DRUG: Inclisiran| BEHAVIORAL: Behavioral Support| DRUG: Background lipid lowering therapy
11	NCT05974345	In Silico Study Assessing the Impact of Inclisiran on Major Adverse Cardiovascular Events in Patients With Established Cardiovascular Disease	SIRIUS/Completed	Study CKJX839B1FR01 in an In silico trial to predict the efficacy of Inclisiran therapy on major adverse cardiovascular events (MACE) and cardiovascular (CV) death in virtual patients with atherosclerotic cardiovascular disease (ASCVD) and elevated LDL-C	Atherosclerotic Cardiovascular Disease	DRUG: Inclisiran sodium| DRUG: Placebo| DRUG: Ezetimibe| DRUG: Evolocumab

Phase	Follow-up duration	Enrollment	Study type	Study design	Locations
PHASE 2	523 days	501	INTERVENTIONAL	Allocation: RANDOMIZED| Intervention Model: PARALLEL| Masking: DOUBLE | Primary Purpose: TREATMENT	Multiple, International
PHASE2	1,695 days	382	INTERVENTIONAL	Allocation: NONRANDOMIZED Intervention Model: PARALLEL| Masking: NONE| Primary Purpose: TREATMENT	Multiple, International
PHASE3	1,399 days	3,275	INTERVENTIONAL	Allocation: NA |Intervention Model: SINGLE GROUP| Masking: NONE| Primary Purpose: TREATMENT	Multiple, International
PHASE3	628 days	1,561	INTERVENTIONAL	Allocation: RANDOMIZED| Intervention Model: PARALLEL| Masking: DOUBLE | Primary Purpose: TREATMENT	United States
PHASE3	637 days	1,617	INTERVENTIONAL	Allocation: RANDOMIZED| Intervention Model: PARALLEL| Masking: DOUBLE/Primary Purpose: TREATMENT	Multiple, International
PHASE2	444 days	312	INTERVENTIONAL	Allocation: RANDOMIZED| Intervention Model: PARALLEL| Masking: QUADRUPLE | Primary Purpose: TREATMENT	Japan
PHASE3	812 days	450	INTERVENTIONAL	Allocation: RANDOMIZED| Intervention Model: PARALLEL| Masking: NONE| Primary Purpose: TREATMENT	United States
PHASE3	330 days	400	INTERVENTIONAL	Allocation: RANDOMIZED| Intervention Model: PARALLEL |Masking: NONE| Primary Purpose: TREATMENT	United States
NA	NA	95	OBSERVATIONAL	Observational Model: |Time Perspective: p	Austria
PHASE3	270 days	892	INTERVENTIONAL	Allocation: RANDOMIZED| Intervention Model: PARALLEL| Masking: NONE| Primary Purpose: HEALTH_SERVICES_RESEARCH	United Kingdom
NA	NA	204,691	OBSERVATIONAL	Observational Model: |Time Perspective: p	France

ASCVD, atherosclerotic cardiovascular disease; NA, low density lipoprotein-cholesterol; ALN-PCSSC/CKJX839B1FR01, Investigational designation of inclisiran

## Results

### Number of studies returned by the search

**FIGURE 1:**
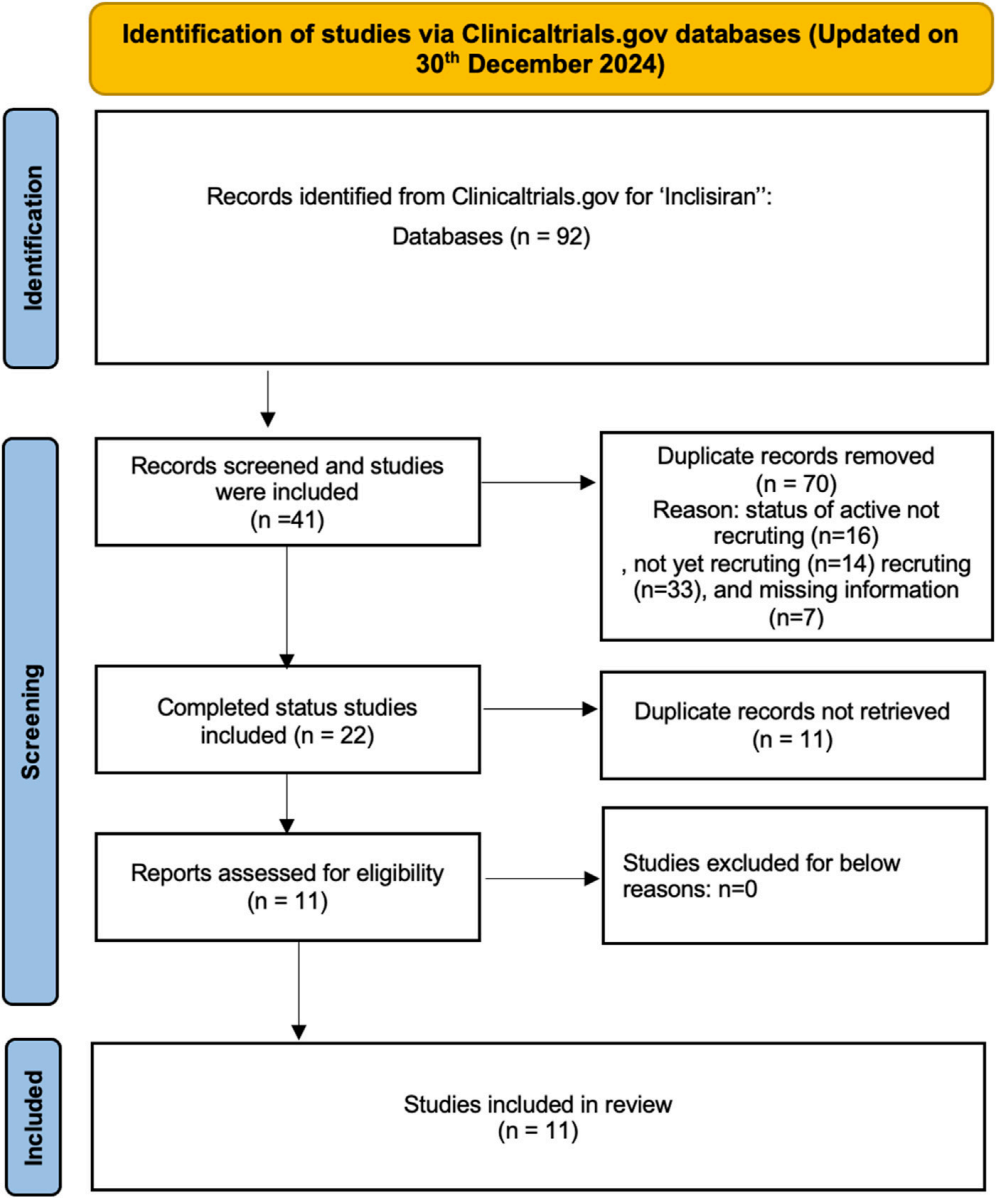
Identification of studies via Clinicaltrials.gov databases.

The preliminary search of the ClinicalTrials.gov database for the “condition/disease” keywords and inclisiran the “other terms” field yielded 92 trials. These trials were extraced and manually examined. After screening the 92 trials, 70 studies were discarded that had a study status of active not recruting, not yet recruting and recruting were excluded. The remaining 41 trials were further examined and 22 completed trials were found but 11 duplicates were removed. Finally, this process yielded 11 completed studies that were included in this review. A synopsis of the study selection process is presented in Figure 1.

### Characteristics of included studies

The included clinical trials mainly examined the effect of inclisiran in patients with cardiovascular diseases. They included a total of 214,176 participants. Most of the studies included were randomized (n = 8). In terms of masking, four of the studies involved either double or quadruple masking, and the remaining studies did not involve any masking due to the study design. Geographically, four of the studies involved multiple western countries with one study conducted Japan. The full details of the included trials are shown in Table 1.

## Discussion

Inclisiran, a first-in-class siRNA-based therapy, represents a novel approach to lipid management by targeting PCSK9 mRNA, enhancing LDL receptor recycling, and providing sustained LDL-C reduction (Do et al., 2013; Fitzgerald et al., 2014). Across pivotal trials, including ORION-9, ORION-10, and ORION-11, inclisiran demonstrated LDL-C reductions of approximately 50%, with effects maintained over extended periods (Ray et al., 2023). These findings highlight its role in addressing unmet needs for hypercholesterolemia management in diverse patient populations, particularly those with atherosclerotic cardiovascular disease (ASCVD) and familial hypercholesterolemia.

### Regulatory status and global adoption

Inclisiran has achieved regulatory approval in multiple countries, reflecting its growing acceptance as a novel lipid-lowering therapy. Initially approved by the European Medicines Agency (EMA) in December 2020, inclisiran is indicated for the treatment of hypercholesterolemia and mixed dyslipidemia in adults who require additional LDL-C lowering despite maximally tolerated statin therapy (Leqvio, 2024). Shortly thereafter, the U.S. Food and Drug Administration (FDA) approved inclisiran in December 2021 as an adjunct to diet and maximally tolerated statin therapy for the treatment of adults with heterozygous familial hypercholesterolemia (HeFH) or ASCVD who require additional lowering of LDL-C (Administration, 2021). Regulatory approvals have also been granted in other regions, including Japan, Canada, and Australia, further expanding its global reach.

### Practical advantages, safety, and adherence

Inclisiran offers significant practical advantages, due in large part to its unique biannual dosing regimen. Administered subcutaneously by healthcare professionals twice yearly following an initial loading dose, inclisiran reduces the treatment burden associated with daily or biweekly dosing (Ray et al., 2020). This dosing regimen aligns with routine clinical visits, thus facilitating regular dosing and significantly improving adherence compared with self-administered therapies such as PCSK9 monoclonal antibodies (Soffer et al., 2022). The SPIRIT trial provided evidence that the integration of inclisiran in primary care improves patient adherence and eases treatment for both patients and healthcare providers (Wilson et al., 2023). Additionally, the targeted mechanism of action of inclisiran limits systemic exposure, thereby improving its safety profile (Wright et al., 2023). Clinical trials consistently show that the most frequent adverse events reported are injection site reactions, which are generally mild and transient (Wright et al., 2021). Longer trials, such as ORION-3 and ORION-8, have confirmed this good safety profile without any significant increase in serious adverse events (Author Anonymous, 2019; Ray et al., 2023). These characteristics make inclisiran especially useful in high-risk populations, such as those with challenges to adherence or who have limited access to health care services (Wright et al., 2023). However, the extended effect may result in persistent unpleasant reactions that are difficult to reverse, as the drug’s activity remains in the absence of early intervention. This feature demands cautious consideration while providing inclisiran, particularly in patients who may be at increased risk of side effects (Mercep et al., 2022).

### Combination use with statins for statin-intolerant patients

Inclisiran complements the existing lipid-lowering therapies, particularly when used in combination with statins. Statins remain a cornerstone in the management of LDL-C levels, with an established history of effectiveness in reducing cardiovascular events. However, not all patients with statin treatment achieve target levels of LDL-C; some are also intolerant, with adverse effects such as muscle symptoms (Serban et al., 2017). Inclisiran closes these gaps effectively, offering a further reduction in LDL-C by almost 50% when used in combination with statins or other treatments as ezetimibe (Ray et al., 2020). Inclisiran is also a potential option for those who cannot tolerate statins, either as monotherapy or in combination with ezetimibe, to achieve substantial reductions in LDL-C levels without the systemic side effects generally associated with statin therapy (Writing et al., 2022; Wright et al., 2023). Real-world settings have confirmed that inclisiran, given biannually, provides consistent LDL-C lowering and adherence, including in high-risk populations (Writing et al., 2022; Naoum et al., 2024). This is of special value for those patients who have trouble following the dosing regimens of other therapies that are taken more frequently. Ongoing studies assessing the combination of inclisiran with various lipid-lowering agents will further define its place in the management of hypercholesterolemia in a variety of patient populations (Wright et al., 2023).

### Comparison with Anti-PCSK9 monoclonal antibodies

Both the PCSK9 monoclonal antibodies (e.g., evolocumab and alirocumab), and inclisiran target the same PCSK9 pathway but but differ significantly in their mechanisms of action, administration schedules, and clinical implications. Inclisiran is an RNA interference technology that silences PCSK9 mRNA (Khvorova, 2017). This mechanism allows twice yearly dosing following an initial loading dose, in contrast to the biweekly or monthly dosing required for monoclonal antibodies directly binding circulating PCSK9 proteins (Chan et al., 2009; Scicchitano et al., 2021). When combined with maximally tolerated statins, self-administered biweekly or monthly anti-PCSK9 monoclonal antibodies reduce LDL-C by approximately 60%, while twice-yearly inclisiran (administered by a healthcare professional) reduces LDL-C by ≈ 50% (Sabatine, 2019; Raal et al., 2020; Ray et al., 2020). Long-term data from ORION-3 confirmed LDL-C reductions were sustained over 4 years (Sabatine, 2019; Raal et al., 2020; Ray et al., 2020; Ray et al., 2023). Both classes show a minor reduction in lipoprotein(a) and triglycerides, but inclisiran shows a modest increase in high-density lipoprotein cholesterol (HDL) (Sabatine, 2019; Raal et al., 2020; Ray et al., 2020). Monoclonal antibodies will be more optimal in cases where a fast reduction of LDL-C levels is required because the effect of them begins immediately after administration. Both drugs are usually well tolerated, but inclisiran is delivered directly, so the possibility of systemic side effects is reduced. All these differences make inclisiran a potential additional or alternative treatment option, especially for patients with indications for long-term, easy control of cholesterol levels.

### Recent studies and real-world evidence

Clinical trials, including the Phase 3 program and related exploratory analyses, have shown compelling evidence supporting inclisiran’s efficacy and safety. However, crucial concerns remain unsolved, notably about the long-term effects on atherosclerosis and cardiovascular outcomes (Wilkinson et al., 2024). Furthermore, the controlled design of these trials does not fully simulate the complexity encountered in daily clinical practice. Notably, the majority of trial participants were White males, indicating a lack of diversity in the study group (Wright et al., 2021). More research is needed to acquire a better knowledge of inclisiran’s long-term effects, cardiovascular benefits, and safety in varied racial, ethnic, and socioeconomic groups.

Several completed and ongoing studies are evaluating the efficacy, safety, and real-world application of inclisiran across various patient populations. ORION-13 and ORION-16 are phase 3 trials assessing inclisiran in adolescents with homozygous familial hypercholesterolemia (HoFH) and HeFH, respectively (Author Anonymous, 2020b; Author Anonymous, 2020a). The VICTORION-PLAQUE trial is studying inclisiran’s impact on atherosclerotic plaque progression (Author Anonymous, 2022). Real-world implementation trials, including VICTORION-INITIATE and VICTORION-INCEPTION, aim to assess inclisiran’s integration earlier in treatment pathways compared to usual care in patients with ASCVD who had not achieved LDL-C levels below 70 mg/dL despite maximally tolerated statin therapy and patients at high risk for a recurrent cardiovascular event in the first year following acute coronary syndrome (ACS), respectively (Knowlton, 2024; Koren et al., 2024). Early real-world studies from the UK and US indicate significant LDL-C reductions (∼49–56%) and favorable tolerability, mirroring clinical trial results, though further observational data are anticipated to confirm these findings in broader clinical settings (Padam et al., 2022; Chiou et al., 2023).

### Cardiovascular benefits

Cardiovascular outcomes trials in patients with established ASCVD have reported a 15%–20% lower risk of major adverse cardiovascular events (MACE) for anti-PCSK9 monoclonal antibodies (Sabatine, 2019). In an exploratory analysis of 3,655 patients (ORION-9, ORION-10, and ORION-11), the addition of inclisiran was linked with a 26% decreased probability of MACE and a lower risk of fatal and nonfatal myocardial infarction compared to placebo. However, these trials were not designed to directly assess MACE as primary endpoints. Additionally, the SIRIUS trial uses computational models to extrapolate the observed LDL-C reductions achieved with inclisiran to predict its potential impact on MACE, offering insights into its long-term cardiovascular benefits in high-risk populations (Angoulvant et al., 2024). Ongoing studies, such as ORION-4, VICTORION-2 PREVENT, and VICTORION-1 PREVENT, are addressing this gap by evaluating inclisiran’s long-term effects on cardiovascular outcomes, including myocardial infarction, stroke, and cardiovascular mortality, in high-risk populations (Novartis, 2018; Author Anonymous, 2021). Despite these promising projections, real-world data and longer-term follow-up are essential to validate these findings and provide conclusive evidence. These studies will also help identify patient subgroups that might derive the greatest benefit. By bridging this evidence gap, ongoing and future trials will solidify inclisiran’s role not just as a lipid-lowering agent but as a critical component of comprehensive cardiovascular risk reduction strategies.

### Cost-effectiveness and economic considerations

The cost-effectiveness of inclisiran remains a critical consideration in its broader adoption across diverse healthcare systems. Its high initial cost presents issues, especially in resource-constrained contexts where affordability and accessibility are crucial (Stoekenbroek et al., 2018). In recent years, there have been various cost-effectiveness studies conducted on inclisiran. The current state of its cost-effectiveness varies based on the geographic region and the price criteria. In the US market it may be considered cost-effective at certain price points, while in China, significant price reductions would be necessary to achieve cost-effectiveness (Desai et al., 2022; Zhou et al., 2024). European healthcare systems have indeed demonstrated positive outcomes for the addition of inclisiran to standard care in the secondary prevention of ASCVD increases life expectancy by 0.199 years and provides an additional 0.159 QALYs (Galactionova et al., 2022; Deaney et al., 2024). As more real-world data becomes available and pricing strategies evolve, the cost-effectiveness profile of inclisiran may continue to change.

### Implications for special populations

Inclisiran has a number of advantages in several special populations due to its efficacy, safety, and very convenient dosing schedule. No dose adjustment is required for patients with mild, moderate, or severe renal impairment, as was shown in the ORION-7 study (Wright et al., 2020). Similarly, no dose adjustment is necessary for the geriatric population or patients with mild to moderate hepatic impairment, though it has not been evaluated in patients with severe hepatic impairment (Dec et al., 2023). Inclisiran reduces LDL cholesterol in patients with HeFH and appears to be promising in rare HoFH, as seen in ORION-2 (Hovingh et al., 2020). In addition, the drug is being evaluated for use in adolescents with hypercholesterolemia; trials are currently ongoing in studies ORION-13 and ORION-16 (Author Anonymous, 2020b; Author Anonymous, 2020a). It has also shown impressive LDL-C reductions in both diabetic and nondiabetic patients (Leiter et al., 2019). Because of the long-acting effect of the drug, administered every 6 months, inclisiran is of particular benefit in such patients who need long-term management of LDL-C with no treatment burden (Ray et al., 2023). These features are advantages and present the promising versatile option of treating hypercholesterolemia across various populations.

### Study limitations and future directions

Despite its promising profile, inclisiran’s current evidence base has limitations. The lack of direct cardiovascular outcome data necessitates reliance on surrogate endpoints such as LDL-C reduction. Real-world studies addressing long-term safety, adherence, and cost-effectiveness in diverse populations are crucial to validate its broad applicability. Additionally, exploring combination therapy with other lipid-lowering agents could optimize cardiovascular outcomes (Raal et al., 2020). Future research should also focus on expanding evidence in underserved populations, including those in low-resource settings, to ensure inclisiran’s benefits are accessible to all. These efforts will refine inclisiran’s clinical utility and solidify its role in the global management of cardiovascular disease.

## Conclusion

The results and outcomes from the clinical trials justify its inclusion into clinical guidelines. The sustained LDL-C lowering and favorable safety profile set inclisiran to become a valuable addition to patient groups with high cardiovascular risk. Its unique mechanism of action and dosing frequency has the potential to change hypercholesterolemia therapy, especially for patients who are statin intolerant or have difficulty achieving LDL-C reduction with current therapy. Its implementation might result in a shift in the approach to controlling cardiovascular risk, particularly in high-risk groups. The potential public health effect of inclisiran is significant. Inclisiran can contribute to lowering the total burden of cardiovascular disease by offering a dependable and patient-friendly choice for LDL-C reduction. This can lead to fewer cardiovascular events and hospitalizations, hence improving public health outcomes and lowering healthcare expenditures.

## References

[B1] Administration (2021). LEQVIO® (inclisiran) injection, for subcutaneous use. U.S. Food and Drug Administration. Available at: https://www.accessdata.fda.gov/drugsatfda_docs/label/2021/214012lbl.pdf (Accessed January 09, 2025).

[B2] Ambrose J. A. Barua R. S. (2004). The pathophysiology of cigarette smoking and cardiovascular disease: an update. J. Am. Coll. Cardiol. 43 (10), 1731–1737. 10.1016/j.jacc.2003.12.047 15145091

[B3] Author Anonymous (2022). A multi-center, randomized, double-blind, placebo-controlled, parallel-group phase IIIb study evaluating the effect of inclisiran on atherosclerotic plaque progression assessed by coronary computed tomography angiography (CCTA) in participants with a diagnosis of non-obstructive coronary artery disease without previous cardiovascular events. VICTORION-PLAQUE. Available at: https://clinicaltrials.gov/study/NCT05360446 (Accessed December 15, 2024).

[B4] Author Anonymous (2023). A double-blind, randomized, placebo- and active-comparator controlled study to evaluate the efficacy of inclisiran as monotherapy in patients with primary hypercholesterolemia not receiving lipid-lowering therapy VictORION-Mono. Available at: https://clinicaltrials.gov/study/NCT05763875 (Accessed December 15, 2024).

[B5] Anand S. S. Hawkes C. de Souza R. J. Mente A. Dehghan M. Nugent R. (2015). Food consumption and its impact on cardiovascular disease: importance of solutions focused on the globalized food system: a report from the workshop convened by the world heart federation. J. Am. Coll. Cardiol. 66 (14), 1590–1614. 10.1016/j.jacc.2015.07.050 26429085 PMC4597475

[B6] Angoulvant D. Granjeon-Noriot S. Amarenco P. Bastien A. Bechet E. Boccara F. (2024). In-silico trial emulation to predict the cardiovascular protection of new lipid-lowering drugs: an illustration through the design of the SIRIUS programme. Eur. J. Prev. Cardiol. 31 (15), 1820–1830. 10.1093/eurjpc/zwae254 39101472

[B7] Author Anonymous (2019). An open-label extension trial of the phase III lipid-lowering trials to assess the effect of long term dosing of inclisiran given as subcutaneous injections in subjects with high cardiovascular risk and elevated LDL-C. Available at: https://clinicaltrials.gov/study/NCT03814187 (Accessed December 15, 2024).

[B54] Author Anonymous (2020a). “Two part (Double-blind inclisiran versus placebo [year 1] followed by open-label inclisiran [year 2]) randomized multicenter study to evaluate safety, tolerability, and efficacy of inclisiran in adolescents (12 to less than 18 Years) with heterozygous familial hypercholesterolemia and elevated LDL-cholesterol (ORION-16)”. Available at: https://clinicaltrials.gov/study/NCT04652726 (Accessed December 15, 2024).

[B55] Author Anonymous (2020b). “Two part (Double-blind inclisiran versus placebo [year 1] followed by open-label inclisiran [year 2]) randomized multicenter study to evaluate safety, tolerability, and efficacy of inclisiran in adolescents (12 to less than 18 Years) with homozygous familial hypercholesterolemia and elevated LDL-cholesterol (ORION-13)”. Available at: https://clinicaltrials.gov/study/NCT04659863 (Accessed December 16, 2024).

[B8] Author Anonymous (2021). A randomized, double-blind, placebo-controlled, multicenter trial, assessing the impact of inclisiran on major adverse cardiovascular events in participants with established cardiovascular disease. VICTORION-2 PREVENT. Available at: https://clinicaltrials.gov/study/NCT05030428 (Accessed December 15, 2024). 10.1016/j.ahj.2026.107493 42203164

[B9] Chan J. C. Piper D. E. Cao Q. Liu D. King C. Wang W. (2009). A proprotein convertase subtilisin/kexin type 9 neutralizing antibody reduces serum cholesterol in mice and nonhuman primates. Proc. Natl. Acad. Sci. U. S. A. 106 (24), 9820–9825. 10.1073/pnas.0903849106 19443683 PMC2682542

[B10] Chiou T. T. Tomasi K. Taub P. R. Wilkinson M. J. (2023). One year experience with inclisiran in an academic lipid clinic. Am. J. Prev. Cardiol. 15, 100567. 10.1016/j.ajpc.2023.100567

[B11] Deaney C. N. Donaldson M. P. Reesby D. M. Scott V. L. Ellis V. Cole G. E. (2024). Retrospective evaluation of LDL-C levels following first treatment with inclisiran as part of secondary prevention ASCVD risk reduction in a real-world primary care setting. J. Prim. Care and Community Health 15, 21501319241236339. 10.1177/21501319241236339

[B12] Dec A. Niemiec A. Wojciechowska E. Maliglowka M. Buldak L. Boldys A. (2023). Inclisiran-A revolutionary addition to a cholesterol-lowering therapy. Int. J. Mol. Sci. 24 (7), 6858. 10.3390/ijms24076858 37047830 PMC10095256

[B13] Desai N. R. Campbell C. Electricwala B. Petrou M. Trueman D. Woodcock F. (2022). Cost effectiveness of inclisiran in atherosclerotic cardiovascular patients with elevated low-density lipoprotein cholesterol despite statin use: a threshold analysis. Am. J. Cardiovasc Drugs 22 (5), 545–556. 10.1007/s40256-022-00534-9 35595929 PMC9468070

[B14] Diez J. Butler J. (2023). Growing heart failure burden of hypertensive heart disease: a call to action. Hypertension 80 (1), 13–21. 10.1161/HYPERTENSIONAHA.122.19373 36082670

[B15] Do R. Q. Vogel R. A. Schwartz G. G. (2013). PCSK9 Inhibitors: potential in cardiovascular therapeutics. Curr. Cardiol. Rep. 15 (3), 345. 10.1007/s11886-012-0345-z 23338726

[B16] Douglas G. Channon K. M. (2014). The pathogenesis of atherosclerosis. Medicine 42 (9), 480–484. 10.1016/j.mpmed.2014.06.011

[B17] Duncan M. S. Freiberg M. S. Greevy R. A. Jr. Kundu S. Vasan R. S. Tindle H. A. (2019). Association of smoking cessation with subsequent risk of cardiovascular disease. JAMA 322 (7), 642–650. 10.1001/jama.2019.10298 31429895 PMC6704757

[B18] Fitzgerald K. Frank-Kamenetsky M. Shulga-Morskaya S. Liebow A. Bettencourt B. R. Sutherland J. E. (2014). Effect of an RNA interference drug on the synthesis of proprotein convertase subtilisin/kexin type 9 (PCSK9) and the concentration of serum LDL cholesterol in healthy volunteers: a randomised, single-blind, placebo-controlled, phase 1 trial. Lancet 383 (9911), 60–68. 10.1016/S0140-6736(13)61914-5 24094767 PMC4387547

[B19] Franklin B. A. Rusia A. Haskin-Popp C. Tawney A. (2021). Chronic stress, exercise and cardiovascular disease: placing the benefits and risks of physical activity into perspective. Int. J. Environ. Res. Public Health 18 (18), 9922. 10.3390/ijerph18189922 34574843 PMC8471640

[B20] Galactionova K. Salari P. Mattli R. Rachamin Y. Meier R. Schwenkglenks M. (2022). Cost-effectiveness, burden of disease and budget impact of inclisiran: dynamic cohort modelling of a real-world population with cardiovascular disease. Pharmacoeconomics 40 (8), 791–806. 10.1007/s40273-022-01152-8 35723806 PMC9300545

[B21] Gareri C. Polimeni A. Giordano S. Tamme L. Curcio A. Indolfi C. (2022). Antisense oligonucleotides and small interfering RNA for the treatment of dyslipidemias. J. Clin. Med. 11 (13), 3884. 10.3390/jcm11133884 35807171 PMC9267663

[B22] Hajar R. (2019). PCSK 9 inhibitors: a short history and a new era of lipid-lowering therapy. Heart Views 20 (2), 74–75. 10.4103/HEARTVIEWS.HEARTVIEWS_59_19 31462965 PMC6686613

[B23] Hovingh G. K. Lepor N. E. Kallend D. Stoekenbroek R. M. Wijngaard P. L. J. Raal F. J. (2020). Inclisiran durably lowers low-density lipoprotein cholesterol and proprotein convertase subtilisin/kexin type 9 expression in homozygous familial hypercholesterolemia: the ORION-2 pilot study. Circulation 141 (22), 1829–1831. 10.1161/CIRCULATIONAHA.119.044431 32479195

[B24] Ickenstein L. M. Garidel P. (2019). Lipid-based nanoparticle formulations for small molecules and RNA drugs. Expert Opin. Drug Deliv. 16 (11), 1205–1226. 10.1080/17425247.2019.1669558 31530041

[B26] Kazemi A. Sasani N. Mokhtari Z. Keshtkar A. Babajafari S. Poustchi H. (2022). Comparing the risk of cardiovascular diseases and all-cause mortality in four lifestyles with a combination of high/low physical activity and healthy/unhealthy diet: a prospective cohort study. Int. J. Behav. Nutr. Phys. Act. 19 (1), 138. 10.1186/s12966-022-01374-1 36384713 PMC9670610

[B27] Khvorova A. (2017). Oligonucleotide therapeutics - a new class of cholesterol-lowering drugs. N. Engl. J. Med. 376 (1), 4–7. 10.1056/NEJMp1614154 28052224

[B28] Knowlton K. U. (2024). Baseline characteristics of participants enrolled in victorion-inception: a randomized study of inclisiran vs. Usual care in patients with recent hospitalization for an acute coronary syndrome. Am. J. Prev. Cardiol. 19, 100756. 10.1016/j.ajpc.2024.100756

[B29] Koren M. J. Rodriguez F. East C. Toth P. P. Watwe V. Abbas C. A. (2024). An “inclisiran first” strategy vs usual care in patients with atherosclerotic cardiovascular disease. J. Am. Coll. Cardiol. 83 (20), 1939–1952. 10.1016/j.jacc.2024.03.382 38593947

[B30] Lagace T. A. (2014). PCSK9 and LDLR degradation: regulatory mechanisms in circulation and in cells. Curr. Opin. Lipidol. 25 (5), 387–393. 10.1097/MOL.0000000000000114 25110901 PMC4166010

[B31] Leiter L. A. Teoh H. Kallend D. Wright R. S. Landmesser U. Wijngaard P. L. J. (2019). Inclisiran lowers LDL-C and PCSK9 irrespective of diabetes status: the ORION-1 randomized clinical trial. Diabetes Care 42 (1), 173–176. 10.2337/dc18-1491 30487231

[B32] Leon-Latre M. Moreno-Franco B. Andres-Esteban E. M. Ledesma M. Laclaustra M. Alcalde V. (2014). Sedentary lifestyle and its relation to cardiovascular risk factors, insulin resistance and inflammatory profile. Rev. Esp. Cardiol. Engl. Ed. 67 (6), 449–455. 10.1016/j.rec.2013.10.015 24863593

[B33] Leqvio (2024). European Medicines agency (EMA). Available at: https://www.ema.europa.eu/en/medicines/human/EPAR/leqvio (Accessed December 28, 2024).

[B34] Lippi G. Sanchis-Gomar F. (2020). Global epidemiology and future trends of heart failure. AME Med. J. 5, 15. 10.21037/amj.2020.03.03

[B35] Mercep I. Friscic N. Strikic D. Reiner Z. (2022). Advantages and disadvantages of inclisiran: a small interfering ribonucleic acid molecule targeting PCSK9-A narrative review. Cardiovasc Ther. 2022, 8129513. 10.1155/2022/8129513 35237348 PMC8853778

[B36] Nabel E. G. (2003). Cardiovascular disease. N. Engl. J. Med. 349 (1), 60–72. 10.1056/NEJMra035098 12840094

[B37] Naoum I. Saliba W. Aker A. Zafrir B. (2024). Lipid-lowering therapy with inclisiran in the real-world setting: initial data from a national health care service. J. Clin. Lipidol. 18 (5), e809–e816. 10.1016/j.jacl.2024.05.003 38908973

[B38] Novartis P. (2018). HPS-4/TIMI 65/ORION-4: a double-blind randomized placebo-controlled trial assessing the effects of inclisiran on clinical outcomes among people with atherosclerotic cardiovascular disease. Available at: https://clinicaltrials.gov/study/NCT03705234 (Accessed December 30, 2024). 10.1016/j.ahj.2026.107546 42567431

[B40] Padam P. Barton L. Wilson S. David A. Walji S. de Lorenzo F. (2022). Lipid lowering with inclisiran: a real-world single-centre experience. Open Heart 9 (2), e002184. 10.1136/openhrt-2022-002184 36600646 PMC9748986

[B41] Raal F. J. Kallend D. Ray K. K. Turner T. Koenig W. Wright R. S. (2020). Inclisiran for the treatment of heterozygous familial hypercholesterolemia. N. Engl. J. Med. 382 (16), 1520–1530. 10.1056/NEJMoa1913805 32197277

[B42] Ray K. K. Troquay R. P. T. Visseren F. L. J. Leiter L. A. Scott Wright R. Vikarunnessa S. (2023). Long-term efficacy and safety of inclisiran in patients with high cardiovascular risk and elevated LDL cholesterol (ORION-3): results from the 4-year open-label extension of the ORION-1 trial. Lancet Diabetes Endocrinol. 11 (2), 109–119. 10.1016/S2213-8587(22)00353-9 36620965

[B43] Ray K. K. Wright R. S. Kallend D. Koenig W. Leiter L. A. Raal F. J. (2020). Two phase 3 trials of inclisiran in patients with elevated LDL cholesterol. N. Engl. J. Med. 382 (16), 1507–1519. 10.1056/NEJMoa1912387 32187462

[B44] Rehman S. Rehman E. Ikram M. Jianglin Z. (2021). Cardiovascular disease (CVD): assessment, prediction and policy implications. BMC Public Health 21 (1), 1299. 10.1186/s12889-021-11334-2 34215234 PMC8253470

[B45] Rimbert A. Smati S. Dijk W. Le May C. Cariou B. (2021). Genetic inhibition of PCSK9 and liver function. JAMA Cardiol. 6 (3), 353–354. 10.1001/jamacardio.2020.5341 33146683 PMC7643040

[B46] Sabatine M. S. (2019). PCSK9 inhibitors: clinical evidence and implementation. Nat. Rev. Cardiol. 16 (3), 155–165. 10.1038/s41569-018-0107-8 30420622

[B47] Same R. V. Feldman D. I. Shah N. Martin S. S. Al Rifai M. Blaha M. J. (2016). Relationship between sedentary behavior and cardiovascular risk. Curr. Cardiol. Rep. 18 (1), 6. 10.1007/s11886-015-0678-5 26699633

[B48] Scicchitano P. Milo M. Mallamaci R. De Palo M. Caldarola P. Massari F. (2021). Inclisiran in lipid management: a Literature overview and future perspectives. Biomed. Pharmacother. 143, 112227. 10.1016/j.biopha.2021.112227 34563953

[B49] Serban M. C. Colantonio L. D. Manthripragada A. D. Monda K. L. Bittner V. A. Banach M. (2017). Statin intolerance and risk of coronary heart events and all-cause mortality following myocardial infarction. J. Am. Coll. Cardiol. 69 (11), 1386–1395. 10.1016/j.jacc.2016.12.036 28302290

[B50] Shapiro M. D. Fazio S. Tavori H. (2015). Targeting PCSK9 for therapeutic gains. Curr. Atheroscler. Rep. 17 (4), 499. 10.1007/s11883-015-0499-4 25712137 PMC5560054

[B51] Soffer D. Stoekenbroek R. Plakogiannis R. (2022). Small interfering ribonucleic acid for cholesterol lowering - inclisiran: Inclisiran for cholesterol lowering. J. Clin. Lipidol. 16 (5), 574–582. 10.1016/j.jacl.2022.06.009 35909047

[B52] Stoekenbroek R. M. Kallend D. Wijngaard P. L. Kastelein J. J. (2018). Inclisiran for the treatment of cardiovascular disease: the ORION clinical development program. Future Cardiol. 14 (6), 433–442. 10.2217/fca-2018-0067 30375244

[B53] Tsao C. W. Aday A. W. Almarzooq Z. I. Alonso A. Beaton A. Z. Bittencourt M. S. (2022). Heart disease and stroke statistics-2022 update: a report from the American heart association. Circulation 145 (8), e153–e639. 10.1161/CIR.0000000000001052 35078371

[B56] Wang N. Tall A. R. (2017). A new approach to PCSK9 therapeutics. Circ. Res. 120 (7), 1063–1065. 10.1161/CIRCRESAHA.117.310610 28264867 PMC5848492

[B57] Wilkinson M. J. Bajaj A. Brousseau M. E. Taub P. R. (2024). Harnessing RNA interference for cholesterol lowering: the bench-to-bedside story of inclisiran. J. Am. Heart Assoc. 13 (6), e032031. 10.1161/JAHA.123.032031 38456415 PMC11010004

[B58] Wilson P. M. Dixon S. Vell T. Bower P. Rootkin L. Williams C. (2023). Implementation of inclisiran in United Kingdom primary care for patients with atherosclerotic cardiovascular disease or its-risk equivalents: rationale and design of Victorion-spirit, a pragmatic phase IIIb, randomised controlled study. Int. J. Clin. Trials 10 (2), 156–165. 10.18203/2349-3259.ijct20231106

[B59] Wong N. D. Shapiro M. D. (2019). Interpreting the findings from the recent PCSK9 monoclonal antibody cardiovascular outcomes trials. Front. Cardiovasc Med. 6, 14. 10.3389/fcvm.2019.00014 30895178 PMC6414420

[B39] World Health Organization (2023). Cardiovascular diseases. Available at: https://www.who.int/health-topics/cardiovascular-diseases#tab=tab_1 (Accessed December 15, 2024).

[B60] Wright R. S. Collins M. G. Stoekenbroek R. M. Robson R. Wijngaard P. L. J. Landmesser U. (2020). Effects of renal impairment on the pharmacokinetics, efficacy, and safety of inclisiran: an analysis of the ORION-7 and ORION-1 studies. Mayo Clin. Proc. 95 (1), 77–89. 10.1016/j.mayocp.2019.08.021 31630870

[B61] Wright R. S. Koenig W. Landmesser U. Leiter L. A. Raal F. J. Schwartz G. G. (2023). Safety and tolerability of inclisiran for treatment of hypercholesterolemia in 7 clinical trials. J. Am. Coll. Cardiol. 82 (24), 2251–2261. 10.1016/j.jacc.2023.10.007 38057066

[B62] Wright R. S. Ray K. K. Raal F. J. Kallend D. G. Jaros M. Koenig W. (2021). Pooled patient-level analysis of inclisiran trials in patients with familial hypercholesterolemia or atherosclerosis. J. Am. Coll. Cardiol. 77 (9), 1182–1193. 10.1016/j.jacc.2020.12.058 33663735

[B63] Writing C. Lloyd-Jones D. M. Morris P. B. Ballantyne C. M. Birtcher K. K. Covington A. M. (2022). 2022 acc expert consensus decision pathway on the role of nonstatin therapies for LDL-cholesterol lowering in the management of atherosclerotic cardiovascular disease risk: a report of the American college of cardiology solution set oversight committee. J. Am. Coll. Cardiol. 80 (14), 1366–1418. 10.1016/j.jacc.2022.07.006 36031461

[B64] Yang H. X. Zhang M. Long S. Y. Tuo Q. H. Tian Y. Chen J. X. (2020). Cholesterol in LDL receptor recycling and degradation. Clin. Chim. Acta 500, 81–86. 10.1016/j.cca.2019.09.022 31770510

[B65] Zhou W. Liang Z. Lou X. Wang N. Liu X. Li R. (2024). The combination use of inclisiran and statins versus statins alone in the treatment of dyslipidemia in mainland China: a cost-effectiveness analysis. Front. Pharmacol. 15, 1283922. 10.3389/fphar.2024.1283922 38469404 PMC10925700

